# The effectiveness of Schroth exercises in adolescents with idiopathic scoliosis: A systematic review and meta-analysis

**DOI:** 10.4102/sajp.v75i1.904

**Published:** 2019-06-03

**Authors:** Marlette Burger, Wilna Coetzee, Lenka Z. du Plessis, Larissa Geldenhuys, Francois Joubert, Elzanne Myburgh, Chante van Rooyen, Nicol Vermeulen

**Affiliations:** 1 Department of Health and Rehabilitation Sciences, Stellenbosch University, Cape Town, South Africa

**Keywords:** adolescent idiopathic scoliosis, Schroth exercises, physiotherapy, Cobb angle, quality of life, systematic review, meta-analysis

## Abstract

**Background:**

Adolescent idiopathic scoliosis (AIS) is one of the most common structural spinal deformities in adolescents, becoming apparent around the time of puberty. Schroth scoliosis-specific exercises have demonstrated promising results in reducing the progression of AIS.

**Objectives:**

The aim of this study was to identify, critically appraise and establish the best available evidence for the effectiveness of Schroth exercises in comparison to non-surgical management to reduce the progression of AIS.

**Methodology:**

Seven databases were searched in April 2018. Main key search terms included *AIS, Schroth exercises, physiotherapy, exercise, electrical stimulation, yoga, Pilates, tai chi* and *bracing*. The quality of the trials was critically appraised according to the PEDro scale. Revman© Review Manager Software was used to pool the quality of life (QOL) results.

**Results:**

Four randomised control trials with an average PEDro score of 6.75/10 were included in this study. Results indicated that Schroth exercises had a significant effect in decreasing the Cobb angle (*p* < 0.05) in comparison to non-surgical management. The pooled effect on QOL showed a significant result in favour of Schroth exercises at 12 weeks (*p* < 0.002) and at 24 weeks (*p* < 0.0004).

**Conclusion:**

Level II evidence suggests that Schroth exercises have a significant effect on reducing the Cobb angle and improving QOL in adolescents with idiopathic scoliosis.

**Clinical implications:**

This review’s findings should be considered with caution for physiotherapy practice because of the limited number of identified articles and their methodologic limitations. Based on the current available and limited evidence, clinicians could combine supervised Schroth exercises with conventional physiotherapy care (observation, exercise, bracing and manual therapy) when treating adolescents with idiopathic scoliosis.

## Introduction

Adolescent idiopathic scoliosis (AIS) is the most common structural spinal deformity occurring in adolescents (Cheng et al. 2017). Adolescent idiopathic scoliosis presents as a laterally rotated curvature of the spine and becomes apparent in generally healthy children around the time of puberty (Weinstein et al. 2008). Adolescent idiopathic scoliosis is diagnosed when the spinal curvature in the coronal plane is equal to or greater than 10 degrees. This spinal curvature is known as the Cobb angle (Cheng et al. 2017), and it is determined by measuring the angle between the upper and lower limits of the deformity in the coronal plane (Morrissy et al. 1990). The magnitude of the Cobb angle is used for classification or to categorise the severity of the scoliosis (Romano et al. 2012). A curve of up to 25° is considered a mild form of scoliosis. Values between 25° and 45° are classified as being a moderate form of this condition and a curve above 45° as severe scoliosis (Romano et al. 2012). If the spinal curve is more than 30° at the end of growth, the potential risks in adulthood increase significantly. These potential risks include pain, deformity of the thorax and shoulder girdle, decreased quality of life (QOL), disability and possible respiratory problems that can progress during adulthood (Altaf et al. 2013; Romano et al. 2012).

Idiopathic scoliosis has traditionally been described as a pain-free condition (Choudhry, Ahmad & Verma 2016). However, a study conducted by Ramirez, Johnston and Brown (1997) found that 31.5% of patients with scoliosis presented with back pain. Patients with idiopathic scoliosis usually only complain of back pain and/or radicular symptoms when they have reached adulthood. Back pain may be the result of spinal imbalance, facet arthropathy, muscle imbalance and fatigue, or foraminal stenosis (Agabegi et al. 2015).

Scoliosis can lead to prolonged incorrect postures and can influence self-image tremendously, especially in the younger female population (Asher & Burton 2006). This in itself can lead to psychological disturbances such as depression (Asher & Burton 2006). Pulmonary impairment manifesting as shortness of breath can be significant in Cobb angles bigger than 80° and in cases where thoracic rotation is present (Asher & Burton 2006). Reduced exercise tolerance, reduced diffusion capacity and a lower maximum oxygen consumption (VO_2_ max) can also be evident in patients with moderate to severe curves (Agabegi et al. 2015). Patients with scoliosis can easily maintain a normal level of functional activity with regard to occupation and family; however, some physical activities, such as lifting, prolonged sitting and standing, and walking long distances can be demanding (Agabegi et al. 2015; Asher & Burton 2006). Therefore, once the diagnosis has been made, effective management should be instituted immediately to address the deformity and to prevent its long-term sequelae.

The non-surgical management of AIS includes observation and conservative treatment. Observation is the first approach to idiopathic scoliosis, and it is performed by regular clinical assessment with a specific follow-up period. When the curvature is less than 25°, the patient is normally observed every 6 to 12 months (Weiss, Weiss & Petermann 2003). The main goals of conservative treatment of AIS are the prevention of curve progression and the cosmetic improvement of the trunk (Vasiliadis & Grivas 2008). The most common interventions used in conservative treatment of AIS are bracing, exercise therapy and manual therapy (Lewis 2012). Bracing is the application of external support to the trunk, and it is used to straighten the spine and de-rotate the pelvis and shoulders to try to achieve normal alignment of the entire body (Rigo et al. 2006). This method of treatment is indicated if the Cobb angle is larger than 25° (Richards et al. 2005). The external and proprioceptive effects resulting from bracing lead to changes in the abnormal loading on the spine and rib cage, reducing asymmetrical movements and increasing neuromuscular control. This facilitates correct spinal growth and neuromotor reorganisation, as well as changes in motor behaviours (Negrini et al. 2010). The outcome of bracing, however, is directly related to compliance, as it should be worn for a prolonged time (minimum of 23 h/day) over several years until skeletal maturity is reached, which generally occurs at 16 years of age for females and 18 years of age for males (Katz & Durrani 2001). Bracing can also be a stressful experience for patients and can worsen self-image and body image, interactions with others and total QOL, which is already impacted by the condition of AIS (Reichel & Schanz 2003). Most braces have the disadvantage of being very bulky and uncomfortable to wear, especially for long periods, and this leads to non-compliance of brace-wear (Canavese & Kaelin 2011).

The overall aim of exercise therapy and specific exercises is to reduce the progression of the scoliotic deformity and to delay or avoid the need of wearing a brace (Romano et al. 2013). Adolescent patients with idiopathic scoliosis with thoracic Cobb angles up to 25° and lumbar or thoraco-lumbar curves up to 20° receive exercises alone, while patients with thoracic main curves between 25° and 50° and lumbar or thoraco-lumbar curves between 20° and 40° receive bracing combined with an exercise regime (Monticone et al. 2014). Exercises to address scoliosis include physical exercises such as strengthening, mobilising, machine-assisted exercises, electrical stimulation, breathing and postural correction exercises (Monticone et al. 2014), as well as low-impact exercises like Pilates, yoga or tai chi to improve trunk flexibility and strength (Blum 2002; Romano et al. 2012).

The Schroth method of exercise is a scoliosis-specific modality used to treat idiopathic scoliosis in young adolescents (Schreiber et al. 2016). In an updated systematic review, Fusco et al. (2011) showed a marked improvement in back strength and breathing function, as well as slowing the rate of curve progression and decreasing the Cobb angle in AIS, when using Schroth exercises. Schroth exercises are described as a ‘method that consists of sensorimotor, postural and breathing exercises aimed at recalibration of normal postural alignment, static/dynamic postural control, and spinal stability’ (Schreiber et al. 2016). In addition to improving various measures of scoliosis, functionality and QOL, Schreiber et al. (2015) reported that adolescents with curves between 10° and 45° responded well to Schroth exercises, resulting in an increase in self-esteem and improved psychological outcomes.

To date, no systematic review comparing the effectiveness of Schroth exercises versus non-surgical management (including observation or conservative management such as the wear of a brace and/or exercise therapy) has been conducted. Several systematic reviews have been published on the effect of exercise in the management of idiopathic scoliosis (Berdishevsky et al. 2016; Romano et al. 2012), but none of them focused on Schroth exercises. Park, Jeon and Park (2017) published a meta-analysis focusing on the effects of Schroth exercise on idiopathic scoliosis; however, they mainly focused on the overall effect size of pre- and post-Schroth exercise subgroup analyses and did not report between-group analyses. The different subgroups were Cobb’s angle ranges, treatment durations and specific Schroth exercises. They found that Schroth exercises practised for at least 1 month were more beneficial for scoliosis patients who had a 10° to 30° Cobb’s angle compared to those with a Cobb’s angle greater than 30°.

The aim of this systematic review was to identify, critically appraise, evaluate and establish best current available evidence on the effectiveness of Schroth exercises to reduce the Cobb angle and improve QOL compared to non-surgical management (including observation or conservative management) in patients with AIS. This will ensure that clinicians have access to the best current available evidence to assist them in the management of adolescents with idiopathic scoliosis.

## Methodology

The Preferred Reporting Items for Systematic Reviews and Meta-Analyses (PRISMA) guidelines were strictly adhered to during the conduct of this systematic review and meta-analysis (Liberati et al. 2009).

### Search strategy

The following seven computerised bibliographic databases, accessed through the Stellenbosch Library services, were searched: PubMed; Science Direct; EBSCOhost: CINAHL and MEDLINE; Cochrane Library; PEDro; Scopus and ProQuest Medical Library. Stepwise documentation of the entire search process was implemented. Key search terms included *AIS, juvenile idiopathic scoliosis, Schroth exercises, physiotherapy, physical therapy, exercise, electrical stimulation, yoga, Pilates, tai chi* and *bracing*. Each database received an individual search strategy according to its function. The databases were divided among the group members, with two authors assigned to each database. The two authors independently searched their allocated database by using the same strategy to ensure proper cross-checking of the results obtained within the different databases. Every database was submitted to the same process and each part of the search process was recorded, documented and cross-checked. Based on the inclusion and exclusion criteria that follow, the authors independently reviewed the titles, abstracts and full-text articles retrieved in the initial search. The authors compared the eligible articles selected for inclusion, and disagreements regarding accepting full-text articles were discussed until consensus was achieved.

### Study inclusion and exclusion criteria

The following inclusion and exclusion criteria were applied.

#### Type of studies

Only randomised controlled trials (RCTs) published in English from inception of the databases until April 2018, scoring at least 4/10 on the PEDro scale (Verhagen et al. 1998), were eligible for inclusion in this review.

#### Type of participants

Study participants could include males and females between the ages of 10 and 19, with a scoliotic curvature of 10° or more (measured by using the Cobb angle). Randomised controlled trials were excluded if they included participants with a scoliotic curvature of less than 10° or if they included participants with scoliosis caused by other pathologies not defined as idiopathic scoliosis.

#### Types of interventions

Studies in which participants received only Schroth exercises as the intervention were included.

#### Types of comparisons

The comparison group had to include participants who received non-surgical management (including observation or conservative management). Conservative management could include the following: wear of brace and/or an exercise regime (including but not confined to stretches, strengthening, mobilising exercises, exercises involving electrical stimulation of specific muscle groups, yoga, Pilates or tai chi).

#### Type of outcomes

Studies that used the Cobb angle as the outcome measure and/or studies that used a QOL questionnaire as the outcome measure were included.

### Evidence hierarchy and methodological appraisal

The relevant articles that met the inclusion criteria for this systematic review were appraised using the National Health and Medical Research Council (NHMRC) hierarchy of evidence (Coleman et al. 2009).

The use of Level II evidence is considered the best for answering intervention questions in a systematic review, whereas Level III and IV evidence becomes progressively less valid and reliable (Coleman et al. 2009). Methodological quality of each included article was appraised using the PEDro scale. The scale appraises internal validity and statistical reporting according to 10 criteria; it is a valid and reliable assessment of the methodological quality of clinical trials (de Morton 2009). Each included article was assigned to two reviewers, who scored the article independently using the PEDro scale. Following this, the two reviewers then compared their scores and, if there were any discrepancies, a third reviewer was consulted. If consensus was still not reached, a group discussion between all eight authors was held to resolve the matter.

### Data extraction and analysis method

The data were extracted and captured on a Microsoft Excel spreadsheet by one author to ensure continuity. Data extracted from the articles included the following categories: citation, study type, participants, interventions, comparisons, outcome measures, results, post-intervention clinical status and implications. The rest of the seven research team members cross-checked the information independently.

Mutual consensus among the group was ensured after a discussion of the complete data extraction. Data pertaining to QOL were synthesised in the form of meta-analyses using the RevMan© Review Manager Software (RevMan© Information Management System 2008), utilising a fixed-effects approach to illustrate combined effects in the form of forest plots. Because the included articles used different versions of the Scoliosis Research Society’s (SRS) health-related QOL questionnaire (https://www.srs.org/professionals/online-education-and-resources/patient-outcome-questionnaires), we used the standardised mean difference in RevMan to convert and standardise the QOL outcomes to a uniform scale. We pooled the means and standard deviations from individual studies to obtain overall estimates and 95% confidence intervals (CIs).

Statistical pooling for the Cobb angle to measure the spinal curvature was considered inappropriate owing to heterogeneity among reporting of results, which were subsequently summarised in a narrative form and illustrated in a table.

### Ethical considerations

This study consists of secondary research, thus ethical approval was not required for this systematic review and meta-analysis.

## Results

### Search results

**FIGURE 1:**
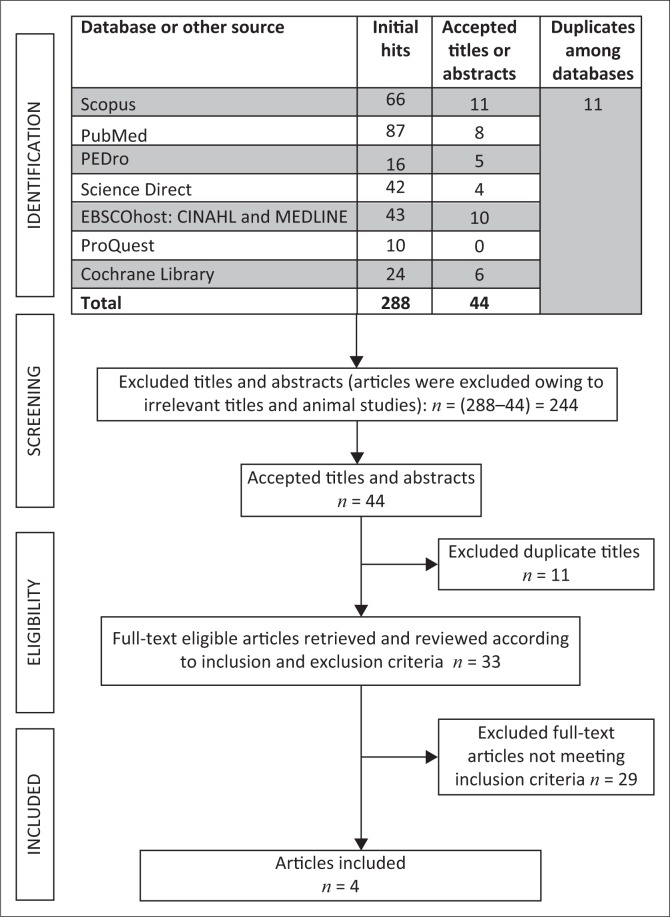
Search results.

A total number of 288 initial hits were found in the seven databases; of these, 244 irrelevant articles were removed. There were 44 remaining potential titles and abstracts reviewed. Out of these 44 titles and abstracts, 11 were excluded because they were duplicates. After removing the 11 duplicate articles, 33 full-text articles remained. These full-text articles were assessed according to the inclusion and exclusion criteria as set out for this study. Twenty-nine full-text articles did not meet the requirements of the inclusion criteria, leaving only four full-text articles suitable to analyse in this systematic review. The search strategy and results are illustrated in Figure 1.

### Evidence hierarchy and methodological appraisal

According to the hierarchy of evidence set forth by the NHMRC (2009), the four included articles (Kim & HwangBo 2016; Kuru et al. 2016; Schreiber et al. 2015, 2016) were classified as Level II. The methodological quality of the four included articles, according to the PEDro scale, ranged between 5/10 to 8/10, with an average score of 6.75/10 (see Table 1). Because of the nature of the included articles, Criteria 4 and 5 (blinding of subjects and therapists) were not met in any of the studies. Kim and HwangBo (2016) and Kuru et al. (2016) also did not blind the assessors in their respective studies.

**TABLE 1:** Study sample description.

Criteria	Variables	Schreiber et al. (2015) and Schreiber et al. (2016)	Kim and HwangBo (2016)	Kuru et al. (2016)
Sample size (*n*)	Schroth exercises	25	12	15
	Home exercises	-	-	15
	Non-surgical management	25	12	15
Gender (*n*)	Schroth exercises	Female = 23 Male = 2	Female = 12 Male = 0	Female = 14 Male = 1
	Home exercises	-	-	Female = 12 Male = 3
	Non-surgical management	Female = 24 Male = 1	Female = 12 Male = 0	Female = 13 Male = 2
Age (years)	Schroth exercises	Mean (range): 13.5 (12.7–14.2)	Mean (SD): 15.6 ± 1.1	Mean ± SD: 12.9 ± 1.4
	Home exercises	-	-	Mean ± SD: 13.1 ± 1.7
	Non-surgical management	Mean (range): 13.3 (12.7–13.9)	Mean (SD): 15.3 ± 0.8	Mean ± SD: 12.8 ± 1.2 (range) (10–18)
Inclusion criteria	Cobb angle	10˚–45˚	≥ 20˚	10˚–60˚
PEDro scores	-	8/10	5/10	6/10

Note: Studies by Schreiber et al. (2015) and Schreiber et al. (2016) were conducted in Canada. Study by Kim and HwangBo (2016) was conducted in Korea. Study by Kuru et al. (2016) was conducted in Turkey.

SD, standard deviation.

### Description of study sample

Table 1 summarises the sample descriptions and interventions of the four included RCTs. All articles had small sample sizes, contributing to a total sample size of 119 participants, consisting of 67 in the intervention groups and 52 in the control groups. Schreiber et al. (2015) and Schreiber et al. (2016) used the same cohort but reported on different outcomes in two papers. All the articles specified gender distribution and had predominantly more female than male participants. The mean age of the participants among the studies ranged between 12.8 and 15.6 years. All the participants were diagnosed with AIS and were otherwise healthy. One of the articles was conducted in an upper-middle income country (Kuru et al. 2016) while the other articles were conducted in high-income countries (Kim & HwangBo 2016; Schreiber et al. 2015, 2016).

### Description of intervention

A comprehensive description of the interventions used across the four articles is outlined in Table 2. Variations in the dosages of Schroth exercises were evident across the four articles. The control groups were exposed to non-surgical management, which involved Pilates exercises, observation or bracing. Kuru et al. (2016) included two intervention groups and a control group. The first intervention group received supervised Schroth exercises, while the second intervention group were taught how to do Schroth exercises under the supervision and guidance of a physiotherapist. The second intervention group were asked to perform the exercises at home, and caregivers were asked to monitor compliance with the home programme.

**TABLE 2:** Description of intervention.

Variable	Description	Schreiber et al. (2015) and Schreiber et al. (2016)	Kim and HwangBo (2016)	Kuru et al. (2016)
Intervention group: Schroth exercises	Method	Supervised Schroth physiotherapeutic scoliosis-specific exercises (individual and group).	Supervised Schroth exercises	Supervised Schroth exercises.
	Dosages and frequency of intervention	Five individual sessions during first 2 weeks for 60 min, followed by weekly 60-min group sessions, in combination with 30–45 min home exercise programme. Total duration of 6 months. Total of 5 individual sessions and 22 group sessions.	Three times a week for 12 weeks for 60 min. Total of 36 sessions	Three days per week for 6 weeks for 90 min. A total number of 18 sessions. The programme was taught to the caregivers as well, and after completion of the 6 week programme, they performed the same programme at home for a 6-month period.
Home exercise group	Method	-	-	Schroth exercises were taught to the subjects under the supervision and guidance of a physiotherapist, and these patients were asked to perform the exercises at home for a 6-month period.
	Dosages and frequency of intervention	-	-	No specific dosages or frequency. Caregivers were asked if exercises were performed regularly to check compliance.
Control group	Method	Observation or bracing in accordance to SRS recommendations.	Pilates exercises	No specific exercises. Only observation.
	Dosage of non-surgical management intervention	SRS recommended dosages.	Three times a week for 12 weeks for 60 min	Participants were observed (assessed) every 6 weeks for a 6-month period.

SRS, Scoliosis Research Society.

### Description of outcome measures and assessment times

The outcome measures and assessment times used in the four articles to assess spinal curvature (Cobb angle) and QOL are shown in Table 3. Standing posterior anterior radiographs were used to measure the Cobb angle in the three included articles (Kim & HwangBo 2016; Kuru et al. 2016; Schreiber et al. 2016). Kuru et al. (2016) and Schreiber et al. (2015) made use of the SRS health-related QOL questionnaire (https://www.srs.org/professionals/online-education-and-resources/patient-outcome-questionnaires). It assesses health-related QOL specific to scoliosis and consists of five domains: function, pain, mental health, self-image and satisfaction with management. The questionnaire has 22 (SRS-22) or 23 items (SRS-23) and a score between 0 and 5 is given to each item, 0 being the worst and 5 being the best (Kuru et al. 2016).

**TABLE 3:** Outcome measures used to measure spinal curvature and quality of life.

Variable	Schreiber et al. (2015)	Schreiber et al. (2016)	Kim and HwangBo (2016)	Kuru et al. (2016)	Assessment intervals
Outcome measure	QOL; SRS-22	Cobb angle	Cobb angle	Cobb angle QOL; SRS-23	Baseline
	-	-	-	QOL; SRS-23	6 weeks
	QOL; SRS-22	-	Cobb angle	QOL; SRS-23	12 weeks
	QOL; SRS-22	Cobb angle	-	Cobb angle QOL; SRS-23	6 months

QOL, quality of life; SRS-22, Scoliosis Research Society 22-item questionnaire; SRS-23, Scoliosis Research Society 23-item questionnaire.

### The effect of Schroth exercises on the Cobb angle and quality of life

The effect of Schroth exercises in the treatment of AIS is shown in Tables 4 and 5 under the following subheadings: Cobb angle and QOL.

**TABLE 4:** Means, standard deviations and *p*-values for Cobb angles measured at baseline, 12 weeks and 24 weeks.

Variable	Description	Baseline		12 weeks		24 weeks	
		Baseline mean ± SD	*p*-value (between groups)	12-week mean ± SD	*p*-value (difference in Cobb angle from baseline between groups)	24-week mean ± SD	*p*-value (difference in Cobb angle from baseline between groups)
Schreiber et al. (2016)	Schroth exercises	29.1 ± 8.9	*p* > 0.05	-	-	27.7 ± 8.9	*p* = 0.006
	Non-surgical management	27.9 ± 8.8		-		29.1 ± 8.8	
Kim and HwangBo (2016)	Schroth exercises	23.6 ± 1.5	-	12 ± 4.7	*p* < 0.05	-	-
	Control group (Pilates exercise)	24 ± 2.6		16 ± 6.9		-	
Kuru et al. (2016)	Schroth exercises	33.4 ± 8.9	*p* = 0.397	-	-	32.3 ± 7.2	*p* = 0.003
	Non-surgical management	30.3 ± 6.6		-		33 ± 6.9	
	Home exercises	30.3 ± 7.6		-		33.8 ± 7.2	

SD, standard deviation.

**TABLE 5:** Means, standard deviations and *p*-values for quality of life measured at baseline, 6 weeks, 12 weeks and 24 weeks.

Variable	Description	Baseline		6 weeks		12 weeks		24 weeks	
		Baseline mean ± SD	*p*-value (between groups)	6-week mean ± SD	*p*-value (between groups)	12-week mean ± SD	*p*-value (difference in QOL from baseline between groups)	24-week mean ± SD	*p*-value (difference in QOL from baseline between groups)
Schreiber et al. (2015)	Schroth exercises	4.25 ± 0.25	-	-	-	4.45 ± 0.25	0.001	4.40 ± 0.25	0.001
	Non-surgical management	4.15 ± 0.25	-	-	-	4.18 ± 0.25		4.15 ± 0.25	
Kuru et al. (2016)	Schroth exercises	3.9 ± 0.6	0.45	4.1 ± 0.5	0.11	4.2 ± 0.5	0.18	4.3 ± 0.5	0.13
	Home exercises	3.9 ± 0.4		4.0 ± 0.5		4.1 ± 0.4		4.1 ± 0.3	
	Non-surgical management	4.1 ± 0.4		4.1 ± 0.4		4.1 ± 0.4		4.0 ± 0.5	

QOL, quality of life; SD, standard deviation.

### Cobb angle

The means, standard deviations (SDs) and *p*-values for Cobb angle measurements are portrayed in Table 4.

Schreiber et al. (2016) measured the largest Cobb angle and found a significant difference (95% CI –5.9° to –1.1°; *p* = 0.006) at 24 weeks in favour of the Schroth exercise group. The largest curve decreased in the Schroth exercise group by 1.2° and increased by 2.3° in the non-surgical management group. Kim and HwangBo (2016) showed a significant decrease in the Cobb angle for both groups (*p* < 0.05) at 12 weeks (intragroup analysis), while the intergroup analysis demonstrated a significant change in favour of the Schroth exercise group (*p* < 0.05). There were significant differences (*p* = 0.003) among the three groups in favour of the Schroth exercise group at 24 weeks in Kuru et al. (2016). The Cobb angle decreased in the supervised Schroth exercise group, while it increased in both the home exercise group and in the non-surgical management group.

### Quality of life

The measurements of QOL are tabulated as the mean and SD in Table 5 using the SRS total score at baseline, 6 weeks, 12 weeks and 6 months. Schreiber et al. (2015) was the only study that found significant differences in favour of the Schroth exercise group at 12 weeks (*p* = 0.0005) and 24 weeks (*p* = 0.001).

**FIGURE 2:**
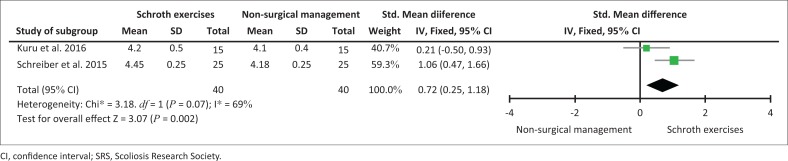
Assessment of quality of life using the SRS-22 and SRS-23 questionnaire after 12 weeks.

**FIGURE 3:**
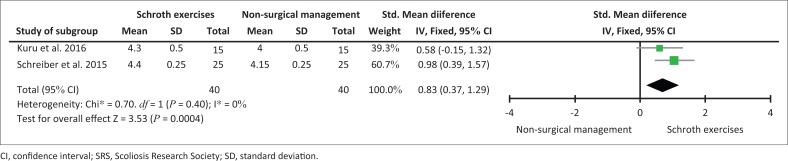
Assessment of quality of life using the SRS-22 and SRS-23 questionnaire after 24 weeks.

When using a meta-analysis to combine the 12-week and 24-week data of Kuru et al. (2016) and Schreiber et al. (2015), the pooled effect showed a significant result in favour of Schroth exercises at 12 weeks (*p* < 0.002) (Figure 2) and at 24 weeks (*p* < 0.0004) (Figure 3).

## Discussion

This is the first systematic review on the effectiveness of Schroth exercises compared to non-surgical management in decreasing the Cobb angle in adolescents with idiopathic scoliosis. Only four RCTs could be found comparing Schroth exercises to non-surgical management after a systematic search of seven databases. Our findings of the evidence suggested that a significant decrease in the Cobb angle can be expected after a 12-week (Kim & HwangBo 2016) and/or a 24-week supervised Schroth exercise programme (Kuru et al. 2016; Schreiber et al. 2016) compared to non-surgical management (see Table 5). A meta-analysis of the overall combined effect (Kuru et al. 2016; Schreiber et al. 2015) indicated that a statistically significant increase in the QOL was found in favour of supervised Schroth exercises at 12 weeks (Figure 2) and 24 weeks (Figure 3).

The treatment frequency and duration of Schroth exercises as a treatment modality differed considerably among the included articles. A meta-analysis conducted by Park et al. (2017) combined 15 studies and measured the pre–post effect size of Schroth exercises on idiopathic scoliosis. The treatment duration was found to be important as the method had a medium effect size (≥ 0.50), according to Cohen’s guidelines (Durlak 2009), if applied for less than 6 months. They recommended that the treatment continue for at least 6 months or longer, as it has been shown to have a large effect size (≥ 0.80) on the Cobb angle. It is interesting to note that the included article with the shortest treatment duration (12 weeks; Kim & HwangBo 2016) also had the largest decrease in Cobb angles in both the Schroth exercise and Pilates groups (see Table 5). This finding is difficult to explain and may be attributed to the higher supervised treatment intensity, namely 36 supervised sessions for a duration of 12 weeks compared to Kuru et al. (2016), who used 18 supervised sessions for 6 weeks, and Schreiber et al. (2016), who included five supervised sessions during the first 2 weeks (see Table 2). Another explanation may be that Kim and HwangBo (2016) also included participants with smaller baseline Cobb angles comparing to Schreiber et al. (2015, 2016) and Kuru et al. (2016) (see Table 4). Park et al. (2017) compared the severity of pre-intervention Cobb angle ranges and reported that as the pre-intervention Cobb angle range increased, the effect sizes decreased. They found that Schroth exercises were much more beneficial for individuals with a pre-intervention Cobb angle range of less than 30° (effect size of ≥ 0.80), whereas Cobb angle ranges of 30°–50° and over 50° showed only a moderate effect size.

Maintaining the improvement reached through supervised exercises with a home exercise programme is essential and the importance of compliance should be emphasised during treatment sessions, especially in countries with limited health resources. Patients who adhere to their prescribed home exercises are significantly better at achieving their goals and demonstrate a greater improvement in physical function (Jack et al. 2010). Unfortunately, research has shown that patients adhere poorly to prescribed home programmes, leading to less favourable functional outcomes (Jack et al. 2010). The success of the Schroth exercises is a responsibility shared by both the physiotherapist and the patient and relies on commitment. Schreiber et al. (2015) had a total compliance rate of 82.5% with regard to the home exercise programme, whereas the attendance rate of the exercise sessions was 85%. The authors attributed the high compliance with the home exercises to the fact that they used exercise logbooks verified daily by the parents and weekly by the therapist (Schreiber et al. 2015). They recommended that compliance with the Schroth home programmes be maximised by providing basic home equipment, parental involvement and keeping a logbook (Schreiber et al. 2015). Kuru et al. (2016) were the only authors that stated that they educated the caregivers to ensure continuity of the supervised Schroth exercise programme as a home exercise programme. Kuru et al. (2016) included a third group, namely an unsupervised Schroth exercise home programme, in their study. The unsupervised home programme group yielded a similar progression and increase in the Cobb angle compared to the non-surgical management group. Kuru et al. (2016) concluded that the capacity for each child to learn and perform Schroth exercises effectively is different and a considerable amount of time should be invested to teach home Schroth exercises to ensure a better outcome. Kim and HwangBo (2016) were the only authors who compared Schroth exercises to Pilates. The exercises that were prescribed in the Pilates group were spinal-correction, balance and core-strengthening exercises with trunk breathing, while the Schroth exercises were prescribed according to the curve shape of each participant. Both Schroth and Pilates showed significant improvement in the Cobb angle; however, the Schroth exercise group proved to be more effective in decreasing the Cobb angle.

The methodological quality of the individual articles ranged from average (5/10) to high (8/10) on the PEDro scale. Because of the nature of the study designs, none of the included studies was able to blind the subjects or the therapists. Kim and HwangBo (2016) and Kuru et al. (2016) did not blind the assessor, and this could have led to observer or detection bias in the individual studies (Hróbjartsson et al. 2012). Kim and HwangBo (2016) did not apply concealed allocation, thus decreasing the internal validity of their study by causing selection bias, which may have influenced the type of treatment that the patients received (Poolman et al. 2007).

The strengths of this review are that a comprehensive and systematic search strategy, including seven computerised scientific databases, was utilised. This review was conducted in a documented stepwise manner. Each step involved two independent authors, and cross-checking was done by the rest of the group, limiting potential errors and encouraging objectivity. A limitation of this review was that only four RCTs met the inclusion criteria. Two of these RCTs used the same cohort (Schreiber et al. 2015, 2016), and both outcomes, QOL and Cobb angle, were only included in one article (Kuru et al. 2016).

Only English papers were included, which could present as possible language bias. However, language translations of studies could not be achieved in the context of this review, as resources and time were limited. Longer-term measurement, including younger adolescents (not at the end of their growth phase), would have strengthened the outcomes.

Higher degrees of scoliosis have many secondary complications (Altaf et al. 2013; Asher & Burton 2006), which necessitates a holistic approach to the management in young adolescents. This review’s positive findings regarding the effectiveness of Schroth exercises in decreasing Cobb angles and improving QOL should be considered with caution for physiotherapy practice because of the limited number of identified articles and their methodologic limitations. Based on the current available and limited evidence, clinicians could combine supervised Schroth exercises with conventional physiotherapy care (observation, exercise, bracing and manual therapy) when treating adolescents with idiopathic scoliosis. It is recommended that a well-structured supervised Schroth home exercise programme be incorporated to optimise outcomes. Schroth exercises do not require expensive equipment, making them very applicable and feasible in the South African context. One of the included articles, with an average PEDro score, found that a decrease in the Cobb angle can be expected after 12 weeks of supervised Schroth exercises, with a frequency of at least three times a week for 60 min (Kim & HwangBo 2016). Three hour-appointments per week for 12 weeks may however be challenging in an under-resourced physiotherapy department and not applicable in the South African context.

Researchers should definitely explore the different domains of QOL, as implemented in Schreiber et al. (2015). Pain, function and self-image are all factors affected by scoliosis, but some to a greater extent than others. It is thus necessary to do further investigation into the outcomes of these individual aspects of QOL and how Schroth exercises affect each of them. This could produce a clearer idea as to where the true improvement lies and also determine what other strategies could be implemented to achieve a better outcome concerning QOL. To determine the long-term effect and sustainability of supervised and home Schroth exercise programmes, long-term studies should be conducted with follow-up assessments at 12 months after completion of the supervised programme. The included studies compared Schroth exercises to non-surgical management, which included bracing and observation as well as Pilates. Because not offering a brace to participants meeting the brace prescription criteria is an ethical concern; Schreiber et al. (2015, 2016) included an equal number of participants (*n* = 17) wearing braces in their Schroth exercise and non-surgical management group. Future studies can look at Schroth exercises combined with brace wearing compared to brace wearing alone and/or observation without brace wearing in participants not yet meeting brace prescription criteria. Because only one of the studies included in this systematic review was not conducted in a high-income country (Kuru et al. 2016), similar studies can be conducted in low or middle-income countries, such as South Africa, to determine the effectiveness of Schroth exercise for adolescents with idiopathic scoliosis in various clinical settings.

## Conclusion

In conclusion, Level II evidence suggests that Schroth therapeutic intervention has a significant effect on improving regression of the Cobb angle as well as improving the QOL in adolescents with idiopathic scoliosis, for up to 6 months post-intervention, when compared to non-surgical management alone.

However, the limited number of identified articles and their methodological shortcomings require caution in interpreting and applying these results. Consequently, further high quality studies should consider the different domains of QOL to evaluate the holistic effect that Schroth exercises can have on the management of scoliosis as well as the long-term effect of Schroth exercises on the Cobb angle.

## References

[CIT0001] Agabegi S., Kazemi N., Sturm P. & Mehlman C, 2015, ‘Natural history of adolescent idiopathic scoliosis in skeletally mature patients: A critical review’, *Journal of the American Academy of Orthopaedic Surgeons* 23(12), 714–723. 10.5435/JAAOS-D-14-00037 26510624

[CIT0002] Altaf F., Gibson A., Dannawi Z. & Noordeen H, 2013, ‘Adolescent idiopathic scoliosis’, *British Medical Journal* 346(1), f2508 10.1136/bmj.f2508 23633006

[CIT0003] Asher M.A. & Burton D.C, 2006, ‘Adolescent idiopathic scoliosis: Natural history and long term treatment effects’, *Scoliosis* 1(2), 1–10. 10.1186/1748-7161-1-2 16759428 PMC1475645

[CIT0004] Berdishevsky H., Lebel V., Bettany-Saltikov J., Rigo M., Lebel A., Hennes A. et al., 2016, ‘Physiotherapy scoliosis-specific exercises – A comprehensive review of seven major schools’, *Scoliosis and Spinal Disorders* 11(1), 1–52. 10.1186/s13013-016-0076-9 27525315 PMC4973373

[CIT0005] Blum C.L, 2002, ‘Chiropractic and pilates therapy for the treatment of adult scoliosis’, *Journal of Manipulative and Physiological Therapeutics* 25(4), E1–E8. 10.1067/mmt.2002.123336 12021749

[CIT0006] Canavese F. & Kaelin A, 2011, ‘Adolescent idiopathic scoliosis: Indications and efficacy of nonoperative treatment’, *Indian Journal of Orthopaedics* 45(1), 7–14. 10.4103/0019-5413.73655 21221217 PMC3004085

[CIT0007] Cheng J., Castelein R., Chu W., Danielsson A., Dobbs M., Grivas T. et al., 2017, ‘Adolescent idiopathic scoliosis’, *Nature Reviews Disease Primers*, viewed 08 October 2017, from http://europepmc.org/abstract/med/27188385. 10.1038/nrdp.2015.30 27188385

[CIT0008] Choudhry M.N., Ahmad Z. & Verma R, 2016, ‘Adolescent idiopathic scoliosis’, *The Open Orthopaedics Journal* 10, 143–154. 10.2174/1874325001610010143 27347243 PMC4897334

[CIT0009] Coleman K., Norris S., Weston A., Grimmer-Somers K., Hillier S., Merlin T. et al., 2009, ‘NHMRC additional levels of evidence and grades for recommendations for developers of guidelines’, *BMC Medical Research Methodology* 9(34), 1–23.19123933

[CIT0010] De Morton N.A, 2009, ‘The PEDro scale is a valid measure of the methodological quality of clinical trials: A demographic study’, *Australian Journal of Physiotherapy* 55(2), 129–133. 10.1016/S0004-9514(09)70043-1 19463084

[CIT0011] Durlak J, 2009, ‘How to select, calculate, and interpret effect sizes’, *Journal of Pediatric Psychology* 34(9), 917–928. 10.1093/jpepsy/jsp004 19223279

[CIT0012] Fusco C., Zaina F., Atanasio S., Romano M., Negrini A. & Negrini S, 2011, ‘Physical exercises in the treatment of adolescent idiopathic scoliosis: An updated systematic review’, *Physiotherapy Theory Practice* 27(1), 80–114. 10.3109/09593985.2010.533342 21198407

[CIT0013] Hróbjartsson A., Sofia A. & Thomsen S, 2012, ‘Observer bias in randomised clinical trials with binary outcomes: Systematic review of trials with both blinded and non-blinded outcome assessors’, *British Medical Journal* 1119, 1–11. 10.1136/bmj.e1119 22371859

[CIT0014] Jack K., McLean S.M., Moffett J.K. & Gardiner E, 2010, ‘Barriers to treatment adherence in physiotherapy outpatient clinics: A systematic review’, *Manual Therapy* 15, 220–228. 10.1016/j.math.2009.12.004 20163979 PMC2923776

[CIT0015] Katz D.E. & Durrani A.A, 2001, ‘Factors that influence outcome in bracing large curves in patients with adolescent idiopathic scoliosis’, *Spine* 26(21), 2354–2361. 10.1097/00007632-200111010-00012 11679821

[CIT0016] Kim G. & HwangBo P, 2016, ‘Effects of Schroth and Pilates exercises on the Cobb angle and weight distribution of patients with scoliosis’, *Journal of Physical Therapy Science* 28(3), 1012–1015. 27134403 10.1589/jpts.28.1012 PMC4842415

[CIT0017] Kuru T., Yeldan İ., Dereli E., Özdinçler A., Dikici F. & Çolak İ, 2016, ‘The efficacy of three-dimensional Schroth exercises in adolescent idiopathic scoliosis: A randomised controlled clinical trial’, *Clinical Rehabilitation* 30(2), 181–190. 10.1177/0269215515575745 25780260

[CIT0018] Liberati A., Altman D.G., Tetzlaff J., Mulrow C., Gøtzsche P.C., Ioannidis J.P. et al., 2009, ‘The PRISMA statement for reporting systematic reviews and meta-analyses of studies that evaluate health care interventions: Explanation and elaboration’, *PLoS Medicine* 6(7), e1000100 10.1371/journal.pmed.1000100 19621070 PMC2707010

[CIT0019] Lewis C, 2012, ‘A review of non-invasive treatment interventions for spinal deformities’, *Physical Therapy Perspectives in the 21st Century – Challenges and Possibilities* 3, 67–88.

[CIT0020] Monticone M., Ambrosini E., Cazzaniga D., Rocca B. & Ferrante S, 2014, ‘Active self-correction and task-oriented exercises reduce spinal deformity and improve quality of life in subjects with mild adolescent idiopathic scoliosis-results of a randomised controlled trial’, *European Spine Journal* 23(6), 1204–1214. 10.1007/s00586-014-3241-y 24682356

[CIT0021] Morrissy R.T., Goldsmith G.S., Hall E.C., Kehl D. & Cowie G.H, 1990, ‘Measurement of the Cobb angle on radiographs of patients who have’, *Journal of Bone & Joint Surgery American Volume* 72(3), 320–327. 10.2106/00004623-199072030-00002 2312527

[CIT0022] Negrini S., Minozzi S., Bettany-Saltikov J., Zaina F., Chockalingam N., Grivas T. et al., 2010, ‘Braces for idiopathic scoliosis in adolescents’, *Spine* 35(13), 1285–1293. 10.1097/BRS.0b013e3181dc48f4 20461027

[CIT0023] National Health and Medical Research Council (NHMRC), 2009, *NHMRC additional levels of evidence and grades for recommendations for developers of guidelines*, NHMRC, Canberra, viewed 01 April 2018, from http://www.nhmrc.gov.au/_files_nhmrc/file/guidelines/stage_2_consultation_levels_and_grades.pdf.

[CIT0024] Park J.H., Jeon H.S. & Park H.W, 2018, ‘Effects of the Schroth exercise on idiopathic scoliosis: A meta-analysis’, *European Journal of Physical and Rehabilitation Medicine* 54(3), 440–449. 10.23736/S1973-9087.17.04461-6 28976171

[CIT0025] Poolman R.W., Struijs P.A., Krips R., Sierevelt I.N., Marti R.K., Farrokhyar F. et al., 2007, ‘Reporting of outcomes in orthopedic randomized trials: Does blinding of outcome assessors matter?’, *Journal of Bone Joint Surgery* 89(3), 550–558. 17332104 10.2106/JBJS.F.00683

[CIT0026] Ramirez N., Johnston C.E.I. & Browne R.H, 1997, ‘The prevalence of back pain in children who have idiopathic scoliosis’, *Journal of Bone and Joint Surgery* 79(3), 364–368. 10.2106/00004623-199703000-00007 9070524

[CIT0027] Reichel D. & Schanz J, 2003, ‘Developmental psychological aspects of scoliosis treatment’, *Pediatric Rehabilitation* 6(3–4), 221–225. 14713589 10.1080/13638490310001644593

[CIT0028] RevMan, 2008, *IMS Cochrane*, viewed 15 June 2018, from http://ims.cochrane.org/revman.

[CIT0029] Richards B., Bernstein R., D’Amato C. & Thompson G, 2005, ‘Standardization of criteria for adolescent idiopathic scoliosis brace studies’, *Spine* 30(18), 2068–2075. 10.1097/01.brs.0000178819.90239.d0 16166897

[CIT0030] Rigo M., Negrini S., Weiss H., Grivas T., Maruyama T. & Kotwicki T, 2006, ‘SOSORT consensus paper on brace action: TLSO biomechanics of correction-investigating the rationale for force vector selection’, *Scoliosis* 1(1), 1 10.1186/1748-7161-1-11 16857045 PMC1553475

[CIT0031] Romano M., Minozzi S., Zaina F., Chockalingam N., Kotwicki T., Hennes A. et al., 2012, ‘Exercises for adolescent idiopathic scoliosis-review’, *Cochrane Database of Systematic Reviews* 8, 10–12. 10.1002/14651858.CD007837.pub2 PMC7386883 22895967

[CIT0032] Romano M., Minozzi S., Zaina F., Saltikov J., Chockalingam N., Kotwicki T. et al., 2013, ‘Exercises for adolescent idiopathic scoliosis’, *Spine* 38(14), E883–E893. 10.1097/BRS.0b013e31829459f8 23558442

[CIT0033] Schreiber S., Parent E., KhodayariMoez E., Hedden D., Hill D., Moreau M. et al., 2016, ‘Schroth physiotherapeutic scoliosis-specific exercises added to the standard of care lead to better Cobb angle outcomes in adolescents with idiopathic scoliosis – An assessor and statistician blinded randomized controlled trial’, *PLoS One* 11(12), 1–17. 10.1371/journal.pone.0168746 PMC5198985 28033399

[CIT0034] Schreiber S., Parent E.C., Moez E.K., Hedden D.M., Hill D., Moreau M.J. et al., 2015, ‘The effect of Schroth exercises added to the standard of care on the quality of life and muscle endurance in adolescents with idiopathic scoliosis – An assessor and statistician blinded randomized controlled trial: SOSORT 2015 award winner’, *Scoliosis* 10(1), 24 10.1186/s13013-015-0048-5 26413145 PMC4582716

[CIT0035] Scoliosis Research Society, viewed 15 June 2018, from https://www.srs.org/professionals/online-education-and-resources/patient-outcome-questionnaires.

[CIT0036] Vasiliadis E. & Grivas T, 2008, ‘Quality of life after conservative treatment of adolescent idiopathic scoliosis’, *Studies in Health Technology and Informatics* 135, 409–413. 18401108

[CIT0037] Verhagen A.P., De Vet H.C., De Brie R.A., Kessels A.G., Broers M., Bouter L. et al., 1998, ‘The Delphi list: A criteria for quality assessment of randomised clinical trials for conducting systematic reviews developed by Delphi consensus’, *Journal of Clinical Epidemiology* 51(12), 1235–1241. 10.1016/S0895-4356(98)00131-0 10086815

[CIT0038] Weinstein S.L., Dolan L.A., Cheng J.C.Y., Danielsson A. & Morcuende J.A, 2008, ‘Adolescent idiopathic scoliosis’, *Lancet* 371, 1527–1537. 10.1016/S0140-6736(08)60658-3 18456103

[CIT0039] Weiss H., Weiss G. & Petermann F, 2003, ‘Incidence of curvature progression in idiopathic scoliosis patients treated with scoliosis in-patient rehabilitation (SIR): An age- and sex-matched controlled study’, *Pediatric Rehabilitation* 6(1), 23–30. 10.1080/1363849031000095288 12745892

